# Genome-wide map of RNA degradation kinetics patterns in dendritic cells after LPS stimulation facilitates identification of primary sequence and secondary structure motifs in mRNAs

**DOI:** 10.1186/s12864-016-3325-7

**Published:** 2016-12-22

**Authors:** Yutaro Kumagai, Alexis Vandenbon, Shunsuke Teraguchi, Shizuo Akira, Yutaka Suzuki

**Affiliations:** 1 Quantitative Immunology Research Unit, WPI Immunology Frontier Research Center, Osaka University, 3-1 Yamada-oka, Suita, Osaka 565-0871 Japan; 2 Immuno-Genomics Research Unit, WPI Immunology Frontier Research Center, Osaka University, 3-1 Yamada-oka, Suita, Osaka 565-0871 Japan; 3 Laboratory of Host Defense, WPI Immunology Frontier Research Center, Osaka University, 3-1 Yamada-oka, Suita, Osaka 565-0871 Japan; 4 Department of Computational Biology and Medical Sciences, Graduate School of Frontier Sciences, The University of Tokyo, Kashiwa, 277-8561 Japan

## Abstract

**Background:**

Immune cells have to change their gene expression patterns dynamically in response to external stimuli such as lipopolysaccharide (LPS). The gene expression is regulated at multiple steps in eukaryotic cells, in which control of RNA levels at both the transcriptional level and the post-transcriptional level plays important role. Impairment of the control leads to aberrant immune responses such as excessive or impaired production of cytokines. However, genome-wide studies focusing on the post-transcriptional control were relatively rare until recently. Moreover, several RNA *cis* elements and RNA-binding proteins have been found to be involved in the process, but our general understanding remains poor, partly because identification of regulatory RNA motifs is very challenging in spite of its importance. We took advantage of genome-wide measurement of RNA degradation in combination with estimation of degradation kinetics by qualitative approach, and performed *de novo* prediction of RNA sequence and structure motifs.

**Methods:**

To classify genes by their RNA degradation kinetics, we first measured RNA degradation time course in mouse dendritic cells after LPS stimulation and the time courses were clustered to estimate degradation kinetics and to find patterns in the kinetics. Then genes were clustered by their similarity in degradation kinetics patterns. The 3′ UTR sequences of a cluster was subjected to *de novo* sequence or structure motif prediction.

**Results:**

The quick degradation kinetics was found to be strongly associated with lower gene expression level, immediate regulation (both induction and repression) of gene expression level, and longer 3′ UTR length. *De novo* sequence motif prediction found AU-rich element-like and TTP-binding sequence-like motifs which are enriched in quickly degrading genes. *De novo* structure motif prediction found a known functional motif, namely stem-loop structure containing sequence bound by RNA-binding protein Roquin and Regnase-1, as well as unknown motifs.

**Conclusions:**

The current study indicated that degradation kinetics patterns lead to classification different from that by gene expression and the differential classification facilitates identification of functional motifs. Identification of novel motif candidates implied post-transcriptional controls different from that by known pairs of RNA-binding protein and RNA motif.

**Electronic supplementary material:**

The online version of this article (doi:10.1186/s12864-016-3325-7) contains supplementary material, which is available to authorized users.

## Background

The expression of genes is regulated at multiple steps in eukaryotic cells, in which the control of RNA concentrations plays a particularly important role. RNA levels are controlled at both the transcriptional level and the post-transcriptional level. There have been large numbers of genome-wide studies on gene expression dynamics focusing on the transcriptional control such as ImmGen [1] and ENCODE [2] projects, while such studies focusing on the post-transcriptional control were relatively rare until recently [3–8].

Immune cells have to change their gene expression patterns dynamically in response to external stimuli such as pathogen associated molecular patterns (PAMPs), which consist of evolutionally conserved bacterial and viral molecular components, including lipopolysaccharide (LPS) consisting of outer layer of Gram-negative bacteria. Both transcriptional and post-transcriptional controls are important in this response [9]. Especially, changes in post-transcriptional control such as RNA degradation are known to be critical for short-term temporal adaptation of gene expression levels [10]. In macrophages and dendritic cells (DCs) various genes including proinflammatory cytokines such as TNF and IL-6 are induced immediately after stimulation by PAMPs. Those genes are also controlled by post-transcriptional mechanisms involving RNA-binding proteins [11]. Several RNA-binding proteins such as HuR [12], AUF1 [13], Zfp36/TTP [14], Arid5a [15], Rc3h1/Roquin [16], Zc3h12a/Regnase-1 [17] have been reported to be involved in post-transcriptional regulation of immune gene expression. Typically, such RNA-binding proteins targets sequence and structural motifs in the 3′ untranslated region (3′ UTR) of RNAs. Deletion of the genes encoding these proteins leads to aberrant immune responses such as excessive or impaired production of cytokines, indicating the importance of such post-transcriptional control.

The next generation sequencing based techniques such as RNA immunoprecipitation sequencing (RIP-seq) or crosslinking and immunoprecipitation sequencing (CLIP-seq) have been used for identification of target motifs of RNA-binding proteins [18]. These methods, which are RNA counterparts of Chromatin immunoprecipitation sequencing (ChIP-seq) to identify *cis* elements of DNA, however, cannot be used if the RNA-binding protein involved in the process of interest is not known. For DNA *cis* elements, an alternative approach for identification starts with the classification of genes according to their gene expression pattern [19]. Typically, gene expression time course data after stimulation is obtained, and genes are classified according to the time course by clustering methods. Subsequently, the promoter sequences of genes with similar expression patterns are subjected to *de novo* motif prediction or detection of known motifs. The advantage of this method is that it does not require any prior information about DNA-binding proteins.

In the current study, we took advantage of the latter approach, starting from classification of genes according to their expression pattern and then leading to *de novo* motif prediction over a set of sequences, to identify RNA *cis* elements possibly controlling RNA degradation kinetics. To classify genes by their RNA degradation kinetics, we first measured RNA degradation time course after LPS stimulation. We found a clear tendency in the kinetics depending on the gene expression levels, induction dynamics after stimulation, and the length of 3′ UTRs. Genes were, then, clustered by their similarity in degradation kinetics patterns. Degradation patterns resulted in a different clustering genes from that obtained from simply gene expression time course profiles. By applying *de novo* sequence or structure motif prediction on the resulting clusters of genes we found not only known functional motifs in 3′ UTRs, but also novel unknown motifs. Together, the current study indicated that degradation kinetics patterns lead to classification different from that by gene expression and the differential classification facilitates identification of functional motifs.

## Methods

### Cells

Bone marrow cells were prepared from C57BL/6 female mice, and were cultured in RPMI 1640 supplemented with 10% of fetal bovine serum under the presence of murine granulocyte/monocyte colony stimulating factor (GM-CSF, purchased from Peprotech) at the concentration of 10 ng/mL. Floating cells were harvested as bone-marrow derived dendritic cells (BM-DCs) after 6 days of culture with changing medium every 2 days. The cells were stimulated with LPS (*Salmonella minnessota* Re595, purchased from Sigma) at the concentration of 100 ng/mL. At 0, 0.5, 1, 2, 3, 4, 6, and 8 h after LPS stimulation, actinomycin D (ActD, from Sigma) was added at the concentration of 10 μg/mL. ActD inhibits transcription and therefore RNA molecules in a cell are degraded over time, which can be measured by RNA-seq to obtain degradation kinetics. At 0, 0.5, 1, 2, and 4 h after ActD addition (40 samples in total), cells were harvested and lysed by TRIzol (Invitrogen). The lysate was further subjected to RNA isolation according to the manufacturer’s instruction. All animal experiments were approved by the Animal Care and Use Committee of the Research Institute for Microbial Diseases, Osaka University, Japan (IFReC-AP-H26-0-1-0).

### RNA sequencing

RNA sequencing was performed as described [20]. The obtained tag sequences were first mapped to rabbit *Hbb2* gene (added as an internal control) and mouse ribosomal RNAs by using Bowtie 2 [21] and the mapped sequences were removed. The remaining unmapped tags were mapped to mm10 genome by using Tophat2 [22]. The number of tags mapped to a gene are counted to obtain tag counts for each transcript. Reads per kilobase per million tags (RPKM) values were calculated for each transcript, then averaged for each gene. Quantile normalization was applied to samples of each time points after ActD addition. The tag sequences in FASTQ format were deposited at DDBJ (accession number DRA004766). The full result is shown in Additional file 1.

### Pattern classification of degradation kinetics

Detailed procedure is described in Additional file 2. Briefly, genes having 1 or more RPKMs in at least one time point before adding ActD were selected as “expressed” genes. The time course data of RNA abundance of these expressed genes after ActD addition were used for pattern classification of degradation kinetics. Time courses with less than 20 tags at time 0 before ActD addition were removed. The remaining time course data were then divided into stable and unstable as follows: if the time course has > 89% (=2^-4/24^; a level which would correspond with a half-life of > 24 h) of the original RNA level at 4 h after ActD addition it was labeled as long-lived. Other time courses were marked as short-lived. Short-lived time course data were clustered by density peak clustering [23] with arccosine of Pearson correlation coefficient as a distance measure for each time course data. The number of clusters was determined as 16 by visual inspection of the *γ*-rank plot, following the guideline in the original paper [23]. For each identified cluster, its degradation rate was calculated by nonlinear fitting [46] to an equation of the form *r*(*t*) = *r*_*1*_ + (*r*_*0*_- *r*_*1*_)exp(-*δt*) with 4 models; *r*_*0*_ = 1 and *r*_*1*_ = 0 with varying *δ* (model 1); *r*_*0*_ = 1 with varying *r*_*1*_ and *δ* (model 2); *r*_*1*_ = 0 with varying *r*_*0*_ and *δ* (model 3); varying *r*_*0*_, *r*_*1*_, and *δ* (model 4). Models were selected according to Akaike’s Information criterion (AIC) [24]. The clusters were ranked by the calculated degradation rates. The calculated degradation rate was also used for visualization and categorization of the genes belonging to the cluster.

### Classification of genes according to degradation patterns

Genes were clustered according to the patterns detected by the above procedure. Genes have 8 degradation patterns (one for each time point after LPS stimulation), which can consist of one of the 16 short-lived patterns, the long-lived pattern, and/or “not applicable” (NA) which means that degradation time course at the time does not fulfill the conditions for further classification as described above. The distance between each pair of patterns is defined as the distance between the means of the corresponding cluster cores. Density peak clustering with this distance measure was performed, and number of clusters was set to nine clusters after visual inspection of *γ*-rank plot. The detailed procedure has been described in Additional file 2.

### Gene ontology analysis

For a given Gene Ontology (GO) term, the number of genes in a cluster or in the whole genome associated with the term were counted. Using the numbers we calculated a hypergeometric *p* values. Benjamini-Hochberg correction was applied to control false discovery rates (FDRs). Subsequent selection of enriched GO terms were performed based on the calculated *q* values with threshold *q <* 0.05.

### *De novo* sequence motif prediction

*De novo* sequence motif prediction was independently performed for each cluster identified according to the degradation pattern. Sequences of 3′ UTRs were extracted from the GenBank RefSeq data (as of April 12, 2015). The sequences are cut into bins with length of 100 bases with 50 bases overlap in-between adjacent bins (see Fig. 5a). For the prediction of enriched primary sequence and secondary structure motifs, we prepared at most 700 sets of sequence bins by randomly selecting single sequence bin for each transcript in the cluster. The following analyses were performed on the sets of sequence bins.

i) **Primary sequence motif prediction**. A *de novo* sequence motif prediction software Weeder (version 1.4) [25] was run over the sets of sequence bins described above. The position frequency matrices (PFMs) of identified motifs were then converted to position weight matrices (PWMs) with pseudocount 0.8 [26]. Based on the PWMs existence of motifs in 3′ UTRs were searched by fimo software from MEME suite (version 4.6.1) [27] with cutoff FDR of 0.05.
ii) **Secondary structure motif prediction**. (1) To identify “seeds” of secondary structure motifs, pairwise alignment considering secondary structure was performed by Foldalign software (version 2.5) [28] over the sets of bins of a cluster of interest. (2) From the identified alignments with various lengths and alignment scores, we chose the “seeds” according to the following three criteria for further analysis: (i) having 15 or more bases in length; (ii) whose sequences are evolutionary conserved, namely, averages of phastCons scores (placental 60-way score downloaded from UCSC genome browser) of both sequences are more than 0.7; and (iii) having scores in the top 0.2% of the set of alignments of each length. Based on the selected alignments, a stochastic context-free grammar (SCFG) model [29] was built by using Infernal software (version 1.1.1) [30]. (4) Existence of the model in the sequences in the cluster was searched by Infernal, and hits with E value more or equal to 1 were selected and aligned. (5) Based on the new alignments, the SCFG model was updated. This motif build, search, and alignment cycle (3–5), was performed until no novel sequences were selected on the searching step. The resulting SCFG models were used as “motifs”, and searched for their existence in 3′ UTRs by Infernal with cutoff E value of 1. Structures of the identified target sequences for motifs were visualized by using RNAfold web server with default parameters and a minimum free energy algorithm (http://rna.tbi.univie.ac.at/cgi-bin/RNAWebSuite/RNAfold.cgi) [31].

### Over-representation analysis of identified motifs

Using the number of hits of the motifs in the genome or a cluster of interest, hypergeometric *p* values were calculated. The calculated *p* values were further subjected to Benjamini-Hochberg correction to obtain *q* values. The significance of the over-representation of motifs in a cluster was determined by the *q* values.

## Results

### RNA-seq measurement of genome-wide RNA degradation kinetics in BM-DC on LPS stimulation

To obtain transcriptome-wide RNA degradation kinetics, we used actinomycin D (ActD) chase experiment procedure. Wild-type BM-DCs were stimulated with 100 ng/mL of LPS for 0, 0.5, 1, 2, 3, 4, 6, and 8 h, and at each time point ActD was added at the concentration of 10 μg/mL. Cells were collected at 0, 0.5, 1, 2, and 4 h after the addition of ActD, resulting in 40 (= 8 × 5) samples in total. Total RNA was prepared and subjected to RNA-seq procedure. The reads were mapped onto mm10 mouse genome, and tag counts and RPKM values for each gene were calculated. This resulted in 13304 genes having 1 or more RPKMs in at least one time point before adding ActD, which we regarded as “expressed” genes.

**Fig. 1:**
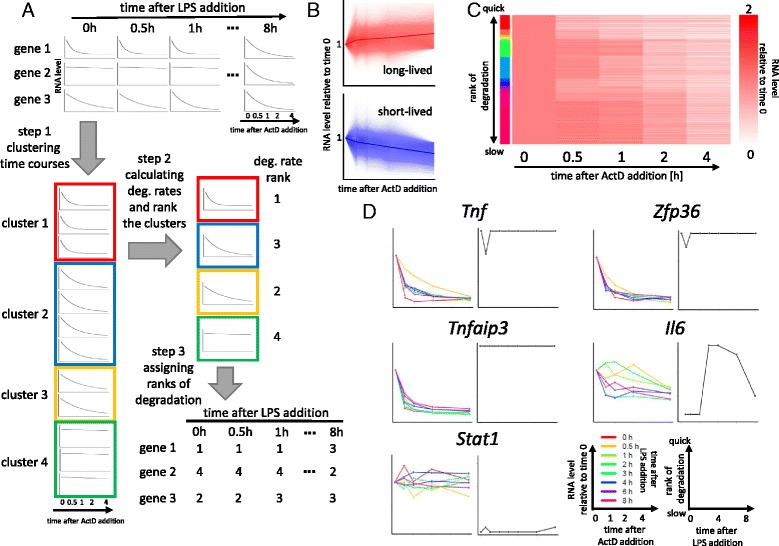
Pattern classification of degradation kinetics. **a** Scheme for classification of degradation patterns. Each gene has 8 time points after LPS stimulation, and 5 time points after ActD addition are associated with each LPS time points. At the first step, these time course data after LPS addition are clustered (Step 1). On the second step degradation rates for each clusters are calculated (Step 2), then assigned the ranks to each genes at each time points after LPS stimulation (Step 3). **b** Time course data classified as long-lived or short-lived. Degradation time course and their means (in *bold line*) were plotted. **c** Heat map image of the resulting clusters. Obtained 16 clusters are indicated by side colors. The clusters are ordered from quickest degradation to slowest from top to bottom. Only cluster cores are shown. **d** Degradation of mRNAs after ActD addition (*left panels*) and rank of degradation rates at each time points after LPS addition (*right panels*) are shown for each genes

The procedure to classify the degradation kinetics of each gene in each duration after LPS stimulation is shown in Fig. 1a. ActD is an inhibitor for RNA polymerase II, thus transcription is supposed to be stopped after the addition of ActD and RNAs would undergo degradation over time. Before proceeding to the classification, degradation time courses with less than 20 tags at time 0 before ActD addition were considered as having too few tags for reliable estimation of degradation parameters, and were therefore marked as “not applicable” and removed. In addition, since we were interested in patters of degradation kinetics of quickly degrading genes, the remaining time course data were then divided into long-lived and short-lived as follows: if the time course has > 89% (= 2^-4/24^; a level which would correspond with a half-life of > 24 h) of the original RNA level at 4 h after ActD addition it was labeled as long-lived. As shown in Fig. 1b, this criterion also separated time courses with increasing signals after ActD addition. In our experimental procedure, RNA levels are measured as relative value to whole RNAs. Since, after addition of ActD, short-lived RNAs decrease while long-lived RNAs remain, resulting in long-lived RNAs appearing to have increasing levels.

Under the assumption that transcripts which degraded with similar kinetics might be regulated by a common mechanism, we first clustered the time courses after the addition of ActD of all genes for each time point after LPS stimulation (step 1 in Fig. 1a). The density peak clustering algorithm was applied to the data and the cluster number was determined as 16, as shown in Fig. 1c and Additional file 3. To link the patterns to degradation speed, we performed nonlinear least-square fitting for each cluster (step 2, Fig. 1a). The degradation kinetics of the unstable RNAs in a cluster was modeled by a differential equation *dr/dt = μ - δr*, where *μ*, *δ* and *r* represent transcription rate, degradation rate, and RNA level, respectively. The relevant model parameters in each cluster were assessed by Akaike’s Information criterion (AIC) as in Methods. The AIC selected the models with incomplete inhibition of transcription for 9 clusters (the model 2 in Methods) and delayed inhibition of transcription for 7 clusters (the model 3 in Methods). Clusters were ranked based on the estimated degradation rates. Cluster images shown in Fig. 1c and Additional file 3 are in the descending order of the rates. Then we assigned the ranks of degradation speed to each time point after LPS stimulation of each gene (step 3, Fig. 1a). The full result was shown in Additional file 4.

We checked the consistency of the estimated degradation kinetics with some representative examples of immune related genes such as *Tnf* and *Zfp36. Tnf* and *Zfp36* mRNAs are known to be stabilized transiently at very early time point after LPS stimulation [32]. Our data recapitulated this transient stabilization of the mRNAs. On the other hand, *Tnfaip3* did not show change in degradation rate, also matching with former studies where the mRNA showed marginal change in degradation rate after stimulation (Fig. 1d, the upper panels and the middle left panel) [33]. Cytokine *Il6* mRNA was destabilized at intermediate time points (3, 4, and 6 h after LPS stimulation; Fig. 1d, the middle right panel), consistent with former results [34]. We also confirmed that interferon-inducible genes like *Stat1* are stable (Fig. 1d, the lowest rows) [35]. Thus, the current procedure successfully recapitulated known patterns of mRNA degradation kinetics.

### Global map of RNA degradation kinetics patterns

**Fig. 2:**
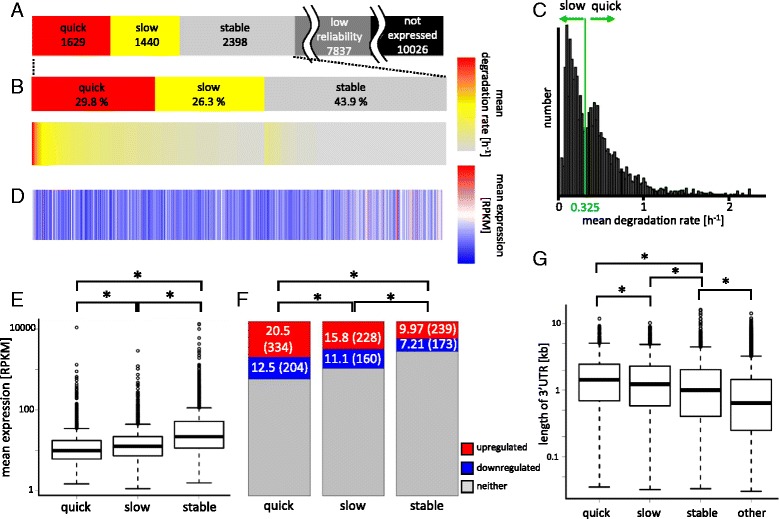
Global map of RNA degradation kinetics. **a** Genes are classified as “not expressed” or “expressed” categories according to expression level. The expressed genes are further categorized according to mean degradation rates, quick, slow, stable, or low reliability, in which calculation of degradation rates of genes were failed at 4 or more time points. Numbers represent number of genes in the category. **b** Genes in quick, slow or stable categories are ordered by mean degradation rates and heat map image of the mean degradation rates is shown. **c** Histogram of mean degradation rates of genes in unstable (quick or slow) category. Genes below or above the green line (degradation rate of 0.325 h^−1^) are categorized as “slow” or “quick”, respectively. **d** Heat map image of mean expression levels of genes are shown in the same order as shown in (**b**). **e** Box plot of mean expression levels for each category. One-sided Mann-Whitney test was performed with Bonferroni correction; *p < 1 × 10^−5^. **f** Bar plot of fractions of up-regulated (*red*), down-regulated (*blue*), or neither (*grey*) genes in each category of degradation. The numbers shown in boxes represent fractions (%), and absolute number of genes in parenthesis. Fisher exact test was performed to compare the numbers of up-regulated genes in two sets with Bonferroni correction; *p < 0.05. **g** Box plot of length of 3′ UTRs of the genes in each category. One-sided Mann-Whitney test was performed with Bonferroni correction; *p < 1 × 10^−3^

Among the 13304 expressed genes, we focused on 5467 genes whose degradation kinetics were reliably identified in 4 or more out of 8 time points after LPS stimulation (Fig. 2a). The mean degradation speeds of the 5467 genes span from a quarter of hour to 24 h with median of 3.8 h in half-life. The 5467 genes were further categorized in the following manner. First, genes labeled as “long-lived” in 4 or more time points after LPS stimulation were categorized as “stable”, and remaining genes as “unstable”, resulting in 2,398 stable and 3,069 unstable genes, respectively. The distribution of the mean degradation rates of the unstable genes was bimodal (Fig. 2c), which motivated us to further categorize them into two classes. Genes degrading more quickly or more slowly than the threshold 0.325 were categorized as “quick” or “slow”, respectively. As shown in Fig. 2a, there were 1629 quick and 1440 slow genes identified.

We next checked how degradation associates with gene expression. Mean gene expression levels were shown in the order of mean degradation rates to compare with each other (Fig. 2b and d). The stable genes tended to have higher gene expression levels than the unstable genes, which was statistically significant as shown in Fig. 2e. Moreover, slow genes had significantly higher gene expression than quick genes (Fig. 2e). Quickly degrading genes and to lesser extent slowly degrading genes were also found to contain significantly higher fraction of upregulated genes than stable genes (Fig. 2f). Also, quickly degrading genes contained higher fraction of upregulated genes than slowly degrading genes did. These results together suggest that quickly degrading genes tend to change their expression level upon stimulation, albeit their mean expression level is lower than others.

The lengths of 3′ UTRs in each category were examined as in Fig. 2g. Quickly degrading genes have significantly longer 3′ UTRs than slowly degrading, stable, and other genes. Similarly, slowly degrading genes tended to have longer 3′ UTRs than stable genes. This implies that quickly degrading genes have longer 3′ UTR which may contain larger number of *cis* elements and may be controlled by multiple mechanisms.

### Functional characters of quick, slow, and stable genes

**Fig. 3:**
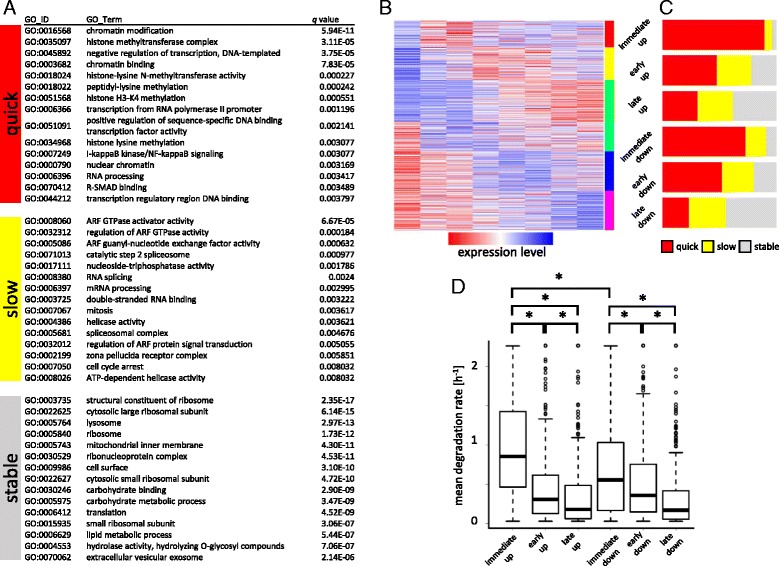
Functional characters of quick, slow, and stable genes. **a** Result of Gene Ontology term enrichment analysis on quick, slow, and stable genes. **b** Heat map image of clusters based on gene expression time course data. The clusters were labeled as “immediate up”, “early up”, “late up”, “immediate down”, “early down”, or “late down” by visual inspection. **c** Bar plot of fraction of quick (*red*), slow (*yellow*) or stable (*grey*) genes in each clusters. **d** Box plot of mean degradation rates of genes in each clusters. One-sided Mann-Whitney test was performed; *p < 1 × 10^−3^

In order to obtain insights into the functions of genes in each category of genes, we performed GO term enrichment analysis. As shown in Fig. 3, quickly degrading genes tend to be associated with epigenetic controls, as well as IKK/NF-κB signaling. Slowly degrading genes are preferentially associated with GTPase regulation as well as control of RNA splicing. In contrast to preferential association with transcription/post-transcription control of the unstable genes, stable genes were associated with translation and metabolism. This result is consistent with former reports where stable genes are more preferentially linked to translation and metabolic processes [36].

LPS stimulation invokes genome-wide change of gene expression levels. We next asked how degradation kinetics contribute to the change in gene expression level. Genes having more than 3-fold up- or down-regulation were selected, and the up- or down-regulated genes were each further classified into 3 clusters (immediate, early, and late) by *k-*means clustering (Fig. 3b). The number of quickly and slowly degrading or stable genes in each cluster were counted (Fig. 3c). Interestingly, sets of immediately up- or down-regulated genes tend to have significantly larger number of quickly degrading genes than the others (*p* < 1 × 10^−4^, Fisher exact test with Bonferroni correction), whereas comparison between immediate up-regulated gene set and down-regulated gene set has no significant change (*p* > 0.05). However, mean degradation rate was significantly higher in immediately up-regulated gene set than in immediately early down-regulated one (Fig. 3d). These results collectively suggested that quick degradation kinetics strongly associated with immediate regulation (both induction and repression) of gene expression level.

### Clusters of genes shared patterns in change of degradation rates over time

*De novo* search of DNA *cis* element is often carried out over a set of genes clustered according to their gene expression time course [19]. This is based on the assumption that the time course represents the change of activity of DNA *cis* elements which control genes in the cluster. For *de novo* identification of RNA *cis* element based on the similar assumption, we first clustered transcripts according to time course of degradation patterns found above.

**Fig. 4:**
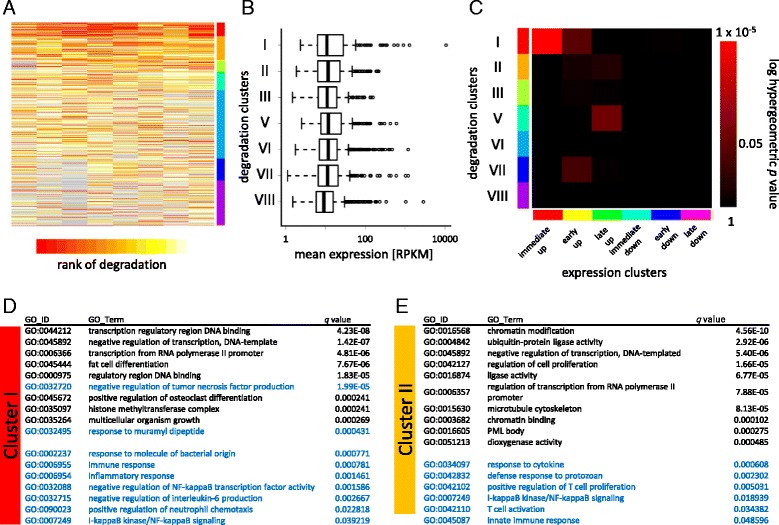
Clustering of genes based on degradation patterns. **a** Heat map image of clusters of genes based on degradation patterns. Color codes represent rank of degradation patterns. Grey represents “NA”. **b** Box plot of mean gene expression levels of genes in each degradation pattern-based cluster (**a**). No significant differences were observed by two-sided Mann-Whitney test with Bonferroni correction (*p* > 0.05), except for cluster VIII compared to clusters I, II, V, VI, or VI (*p* < 0.05) (**c**) Overlaps between the degradation pattern-based clusters (**a**) and the gene expression-based clusters shown in Fig. 3b. Hypergeometric *p* values of overlaps are shown. **d**, **e** Gene Ontology term enrichment in clusters I (**d**) and II (**e**). GO terms in *blue* letters represent immunity-related ones. Top 10 GO terms and significant (*q* < 0.05) immunity-related terms are shown

We separated genes having more than 4 “long-lived” time points as stable genes as described above. From the remaining 3067 unstable genes, the density peak clustering gave nine clusters (Fig. 4a and Additional file 2). Together with transcripts labeled as stable, this resulted in 10 clusters (full result is in Additional file 4). The clusters were further ordered by mean degradation rates of genes in the clusters, from quick to slow, and named as Cluster I, II, … IX, and stable (Fig. 4a). The clusters contain more than hundreds of genes with exception of Clusters IV and IX, containing small number of genes. We excluded these two clusters from further analysis.

First, since we have shown that degradation is associated with gene expression level, we compared the mean expression levels of genes in each cluster. As shown in Fig. 4b, mean gene expression levels were comparable between all clusters, without significant differences (*p* > 0.05, after Bonferroni correction), except for cluster VIII compared to clusters I, II, V, VI, or VI (*p* < 0.05). This indicated that although mean degradation rate is strongly associated with mean expression level, the classification based on the degradation kinetic patterns gives unique clustering of genes different from that on gene expression level. The clusters were further compared to gene expression change-based clusters obtained in Fig. 3b. Hypergeometric *p* values of overlap between two clusters were calculated and shown in Fig. 4c. Cluster I, which contains large number of quickly degrading genes, has significant overlap with immediately up-regulated gene set (*p <* 3 × 10^−5^), further supporting quickly degrading genes are more likely to be up-regulated at early time point after LPS stimulation. The other significant overlap was found only between Cluster V and late up-regulated gene (*p <* 0.05), suggesting that time course of change in degradation pattern offers different classification than gene expression time course or mean degradation rate does.

Cluster I contains numbers of well-known quickly degrading genes such as *Tnf, Zfp36, Tnfaip3*and *Nfkbia* [35]. Gene Ontology term enrichment analysis showed that the cluster is associated with transcriptional regulation and immune responses (Fig. 4d). The cluster also contains *Ppp1r10, Ier3* and *Nfkbid* as well as *Tnf*, known targets for RNA-binding proteins Roquin and Regnase-1, implying transcripts in the cluster are regulated by similar mechanism involving Roquin and Regnase-1 [37, 38].

Cluster II is preferentially associated with chromatin modification genes such as *Kdm6b* and *Setdb1,* ubiquitin ligases, and to lesser extent immune response genes, such as *Il6, Nfkbiz, Nfkb1,* and *Irak3. Il6* and *Nfkbiz* are also known targets for Roquin and Regnase-1 [37, 38], suggesting genes targeted by same RNA-binding proteins do not necessarily share a pattern of degradation kinetics, and regulatory mechanism of degradation of the genes in the cluster is rather heterogeneous (Fig. 4e).

### De novo prediction of primary sequence motifs in 3′ UTRs

**Fig. 5:**
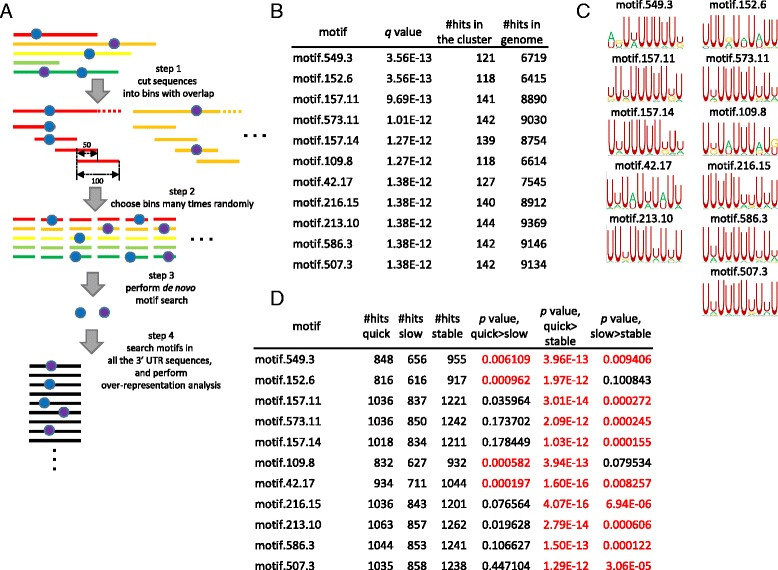
*De novo* sequence motif prediction. **a** Scheme for *de novo* motif prediction. The sequences of 3′ UTR were cut into 100 bases long bins with 50 bases overlap in between adjacent bins (Step 1). From each gene one bin is randomly selected and sets of bins from each gene were randomly generated for hundreds of times (Step 2). *De novo* motif prediction was performed over the sets (Step 3). Motifs found are then subjected to over-representation analysis (Step 4). **b** The resulting over-represented motifs in Cluster I and (**c**) their sequence logos. **d** Number of hits in quick, slow and stable gene sets. Hypergeometric *p* values with Bonferroni correction are also shown. *Red* letters represent significant (*p* < 0.01) differences

Stability of mRNAs is thought to be controlled largely through their 3′ UTRs. Indeed, several primary sequence motifs in 3′ UTR, such as AU-rich elements (AREs) [39], are known to control mRNA stability. Thus it is natural to apply *de novo* prediction of sequence motifs to 3′ UTR sequences of genes in a cluster thought to be controlled by similar degradation mechanism. To test if the current classification of genes based on degradation kinetics patterns helps the identification of sequence and secondary structure motifs *de novo*, we first searched for primary sequence motifs on 3′ UTRs of the transcripts in Cluster I obtained above, since this cluster is enriched for immune related genes. The procedure is shown in Fig. 5a. First we cut the 3′ UTR sequences into 100 base long bins with 50 bp overlap between adjacent bins. Those bins were randomly picked from each gene to make a set of 3′ UTR sequence bins in the cluster. Sets were randomly generated for hundreds of times, and on each set we used the *de novo* sequence motif prediction program Weeder. After identifying motif candidates, their over-representation in the cluster was assessed by calculating FDRs.

A list of enriched motifs found in Clusters I is shown in Fig. 5b and c, respectively. The identified motifs were largely similar to each other. *Tnf*, *Zfp36*, and other genes are known to be regulated through AREs in their 3′ UTRs [39]. Typical ARE has AUUUA pentamer, with some variation in franking nucleotides [40]. As expected from the presence of *Tnf* and *Zfp36* in the cluster, the enriched motifs in the cluster are abundant in U and to lesser extent A. Furthermore, some of the motifs such as motif.109.8 and 42.17 contain AUUUA-like stretch (Fig. 5c), fitting with the consensus motif of AREs. Moreover, these motifs resemble typical TTP binding motif UAUUUAU [41], suggesting the control on number of genes in the cluster by TTP. Finally, we checked the number of presences of these motifs in quick, slow or stable gene sets, and found that the set of quickly degrading genes has significantly higher numbers of instances than the sets of slowly degrading genes or stable genes (Fig. 5d). Collectively, *de novo* prediction of sequence motifs in genes sharing the same degradation pattern identified ARE-like motifs, which are abundant in 3′ UTR of quickly degrading genes.

### De novo prediction of secondary structure motifs in 3′ UTRs

Unlike DNA, most cellular RNAs are single-stranded and form secondary structure, such as stem loop structure. Typical examples are transfer RNA and ribosomal RNAs, which form large secondary structure, and form complex with proteins. For the interaction between RNAs and proteins, such secondary structures are essential. Previous studies have identified various secondary structure motifs bound by certain RNA binding proteins, such as above mentioned RNA-binding proteins [37, 38, 41–43]. Their identification, however, has been based on the prior knowledge that particular RNAs are targets of a particular RNA-binding protein. Since there are hundreds of RNA-binding proteins, it is favorable to identify RNA motifs even when there is no such prior knowledge. Thus, we further sought to identify secondary structure motifs enriched in the cluster in addition to the sequence motifs.

Several methods to extract secondary structure motifs from a set of transcript sequences have been proposed. Some studies took advantage of pairwise or multiple alignment of RNA sequences considering both sequence similarity and secondary structure [43, 44]. We utilized a similar approach with modifications to detect motifs that are enriched in a cluster of interest, but not necessarily form a majority in the cluster. The latter point is crucial since we are not starting from a set of transcripts identified as targets of a common RNA-binding protein, but from a set of transcripts which share only degradation kinetics.

**Fig. 6:**
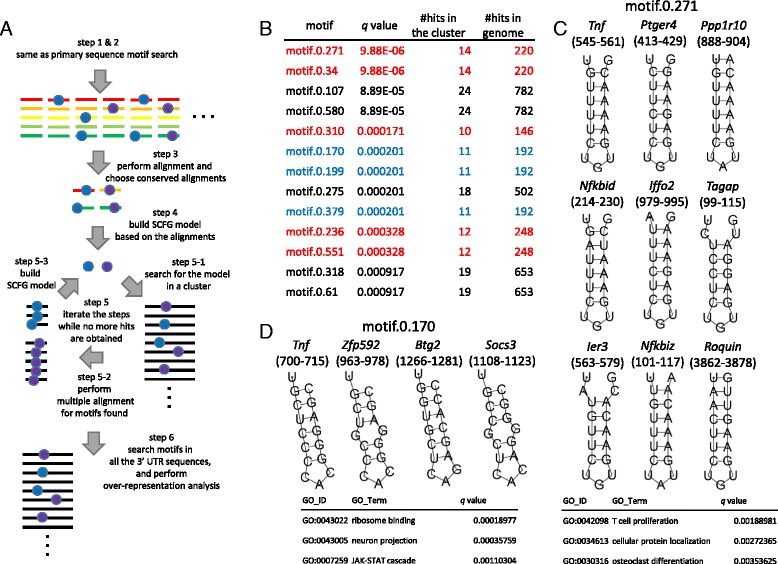
*De novo* secondary structure motif prediction. **a** Scheme for the *de novo* secondary motif prediction. The search was done over the sets of bins generated as in Fig. 5a, Step 1 and 2. Pairwise alignment considering secondary structure was performed on the sets of bins, and conserved and highly scored alignments were selected (Step 3). Using the alignments as “seeds”, SCFG model was generated (Step 4). Motifs were searched in all the 3′ UTRs in the clusters (Step 5-1), then hits were aligned (Step 5-2) and new SCFG model was generated (Step 5-3). This cycle was performed until no new hits were obtained in the searching step. **b** The resulting over-represented motifs in Cluster I. Motifs with *q* < 1 × 10^−4^ and 10 or more hits in the cluster were shown. Motifs in red letters, those in blue letters, or those in black letters represent similar motifs, respectively. **c** Representative structures of motif.0.271 and the top 3 of preferentially associated GO terms in genes having the motif. **d** Representative structure of motif.0.170 and the top 3 of preferentially associated GO terms in genes having the motif

The procedure is briefly depicted in Fig. 6a. We first carried out pairwise sequence and structure alignment of RNAs on 3′ UTR sequence bins. From Cluster I, over 6 million of alignments were found, which are regarded as “seeds” for motifs. We checked the distribution of scores and lengths of the alignments (Additional file 5) and found that they are not independent. Thus, we first divided the alignments into sets of the same length. Then motif seeds were selected from each sets with the following criteria: (1) Aligned sequences are conserved, namely, have more than 0.7 mean phastCons score, and (2) alignment score in the top 0.2% of the set with the same alignment length. After this selection we retained 679 motif seeds from this cluster. Stochastic context free grammar (SCFG) models [29] were built from the selected motif seeds as described in the Methods, and over-representation in the cluster was checked. As shown in Fig. 6b, we obtained 13 motifs with less than 1.0 × 10^−4^ FDR and 10 or more instances in the cluster. Roughly, they were divided into 3 different motifs (red, black and blue in Fig. 6b). All the alignments with common structure for motif.0.271, motif.0.170, and motif.0.107 are shown in Additional file 6. Among the motifs, motifs 0.271, 0.34, 0.310, 0.236 and 0.551 were found to be similar to the stem-loop motif bound by Roquin- or Regnase-1 (Fig. 6b, in red). As shown in Fig. 6c, *Tnf, Ier3, Ppp1r10* and *Nfkbid* were found to contain the motif, and the stem loops are almost identical to that of Roquin or Regnase-1 targets [37, 38]. Other members in the Cluster I, such as *Ptger4*, *Iffo2*, *Tagap*, as well as known Roquin or Regnase-1 targets in other clusters such as *Nfkbiz* and *Roquin*, also have the stem loop. This indicates that the current procedure in combination with genome-wide measurement of RNA degradation kinetics successfully identified functional secondary structure RNA motif without prior information.

In addition to this known functional motif, other stem loop motifs were identified in the current cluster. Motifs indicated in black in Fig. 6b, including motif.0.107 and 0.580, have many AU repeats (Additional file 6), and it is not clear if they represent functional protein binding motifs. Motif.0.170, 0.199, and 0.379 (shown in blue letters in Fig. 6b), on the other hand, were not identical to the Roquin-binding stem loop and did not have repeat sequences. As shown in Fig. 6d, *Tnf*, *Zfp592, Btg2,* and *Socs3,* contain this stem loop structure. We checked GO term enrichment for genes having motif.0.271 or motif.0.170 (tables in Fig. 6c and d, respectively), and found genes with motif.0.271, Roquin and Regnase-1 target, were associated with terms such as T cell proliferation, while those with motif.0.170 were preferentially associated with terms such as JAK-STAT cascade, further implying the role of those motifs in immune responses. These results collectively suggest that degradation patterns of genes in this cluster is regulated by mechanisms which are different from those controlled by Roquin and Regnase-1, and involve the binding of a regulator to this stem-loop structure.

## Discussion

In the current study we conducted genome-wide measurement of RNA degradation kinetics, and used the dynamical pattern in degradation rates for predicting sequence and structure motifs of genes under the similar regulatory mechanism. A number of genome-wide RNA degradation measurements have been reported before [3–8]. Despite cell types and methods being differed between these studies, the reported range of half-life is consistently from half an hour to 8–30 h, with median values of around 1.5 to several hours. While many of such studies focused to estimate degradation rates of genes in a single condition, we aimed to identify the change of degradation rates as a time course after stimulating cells. Although we lacked replicates in our data due to resource limitation, we attempted to circumvent the limitation of quantitative accuracy of the estimated degradation rates by a qualitative approach based on clustering. Namely, we first classified the degradation time courses and then calculated degradation rates for the resulting clusters of time courses. Despite the limitation, the approach led to results consistent with those previous studies. Our result showed that range of half-life is from around a quarter of hour to 24 h with median of 3.8 h, indicating reasonable estimation of the RNA degradation kinetics with our procedure.

It has been suggested that degradation of mRNA of genes depends on their cellular functions [36]. Our result confirmed that mRNAs of house-keeping genes are degraded slowly, while those of genes involved in transcriptional regulation and immune signaling, and those of genes encoding cytokines are degraded quickly. Moreover, since our data contains dynamics of gene expression after LPS stimulation, we could reveal associations between expression patterns and degradation kinetics. For example, genes with immediate changes (both induction and repression) in their expression level after LPS stimulation are degraded quickly. In one of the preceding studies, similar to ours, Rabani et al. [5] measured changes of the half-lives of RNAs after LPS stimulation at genome-wide level. Although they only reported gene expression and degradation kinetics of relatively early time points (up to 3 h after stimulation), they also concluded that immediately and transiently induced genes have high degradation rates. Hao and Baltimore reported consistent results on a smaller set of selected genes [35]. Together, we have shown that degradation kinetics of genes correlate with their physiological function and pattern of gene expression level changes.

Some of aforementioned studies utilized ActD chase experiments for measuring degradation as we did in the current study, while others utilized chemical labeling of RNAs with nucleotide analogs [45]. Tani et al. [8] showed that ActD slowed down the degradation of a large fraction of non-coding RNAs. Indeed, we found many genes, including non-coding RNAs, were classified as “stable”. Furthermore, in our procedure, the degradation rates of many genes could not be calculated. Together, it is possible that the distribution of degradation rates in cells not perturbed with ActD is different from the current result. Nevertheless, we observed a general consistency with former results on a genome-wide level as discussed above.

Immune cells induce or repress many genes after stimulation. RNA degradation is suggested to be important for controlling this drastic change in expression [39]. Several RNA *cis* element and RNA-binding proteins have been found to be involved in the process, but our general understanding remains poor. One reason is that the identification of regulatory RNA motifs is very challenging. The prediction of RNA motifs is therefore of great interest. Typically, motif prediction starts from a set of sequences of which all or a large fraction are expected to share a common motif. Most RNA motif prediction approaches are designed for cases where such prior information is available. Therefore, identification of such RNA motifs has required particular RNA-binding protein specific information obtained by using RIP-seq data [37, 38, 41, 43]. Instead, in the current study we first performed pairwise alignment considering both sequence and structural similarity. We built SCFG models based on the alignments and iteratively refined the models within a cluster of interest. This procedure can successfully find motifs even if they are not abundant in the cluster, and thus it is advantageous for the current case in which genes in a cluster share degradation kinetics patterns but do not necessarily share a common motif. By using this procedure, we started from a classification of genes based on degradation patterns and found known functional RNA motifs, and also predicted new candidate motifs, without prior information of binding proteins, although further experimental verification is needed to ensure the function of the motifs.

The successfully identified model of Roquin-and Regnase-1-binding motifs, which was build based on a cluster of interest, also identified the stem loop motif in genes in other clusters, such as *Nfkbiz* and *Roquin*, suggesting that the model generated by the procedure has generality for finding targets at a genome-wide level. However, the same model failed to identify some of known target genes of the RNA-binding proteins, such as *Il6* and *Zc3h12a/Regnase-1* [34, 37, 38]. Since the current procedure builds models by an iterative search-alignment-build cycle in a cluster, it is presumable that the motif model is biased by sequences in the cluster. Alternatively, this finding might suggest the existence of some variety in the motifs recognized by Roquin and Regnase-1.

## Conclusion

The current study presents a comprehensive map of patterns of RNA degradation kinetics and indicates that the map facilitated *de novo* RNA motif prediction. We found strong association between degradation patterns and expression dynamics. Based on the degradation patterns we could find, not only known functional sequence and secondary structure motifs, but also unknown motifs in 3′ UTR RNA sequences. The result implied existence of post-transcriptional controls different from that by known pairs of RNA-binding protein and RNA motif. The analysis scheme presented in this study is applicable to other types of genome-wide degradation data and will contribute to elucidating the biology of gene expression control, especially at the post-transcriptional level.

## Additional files

Additional file 1: Tag counts and quantile-normalized RPKM values. (XLSX 14403 kb)
Additional file 2: Supplementary Methods. (PDF 514 kb)
Additional file 3: Pattern recognition of degradation kinetics. Time course data in each of 16 clusters with the mean of the core data of the cluster (black dashed line) and fitting curve (red line) were plotted and shown. The clusters are in the descending order of degradation rates calculated from the fitting, from top, left to right, to bottom. (PDF 443 kb)
Additional file 4: Ranks of degradation speed and clusters. (XLSX 924 kb)
Additional file 5: Scatter plot of alignment scores vs alignment lengths obtained from Cluster I. Scores and lengths of conserved alignments from Cluster I were plotted and shown. (PDF 238 kb)
Additional file 6: Multiple alignment of sequences found with each motif. Sequences found by searching with the motifs were aligned by Infernal and shown in Stockholm format. Refseq ID of the origin of the sequence, positions of the 3′ UTR in the mRNAs of the Refseq ID, and positions of the sequence found by motif searching was shown in the left column. Common secondary structure (SS_cons) and consensus sequence (RF) are also shown. (PDF 214 kb)
