# Supplementary material for: Genome-wide map of RNA degradation kinetics patterns in dendritic cells after LPS stimulation facilitates identification of primary sequence and secondary structure motifs in mRNAs
Source: BMC Genomics. 2016 Dec 22;17(Suppl 13):1032. doi: 10.1186/s12864-016-3325-7 (PMC5259865; doi:10.1186/s12864-016-3325-7)
Supplement: Additional file 2: — Supplementary Methods. (PDF 514 kb) [file 12864_2016_3325_MOESM2_ESM.pdf]

Supplementary Methods.

Detailed procedure for the calculation of degradation rates and classification of genes according to the time course of degradation rates.

1) Clustering of time course data of RNA degradation dynamics.

Tag counts were calculated as described in Materials and Methods. For genes having multiple isoforms, tag counts were averaged. Out of 23330 genes in total, genes with RPKM more than 1 in at least one time point before adding ActD were regarded as "expressed" and selected, resulting in the list of 13304 genes.

Each gene has 8 time points after LPS addition, thus there are 13304 x 8 = 106432 of time courses data having 5 time points, 0, 0.5, 1, 2, and 4 hours after ActD addition. To calculate degradation rates from the time course data, we performed clustering and then non-linear fitting, instead of performing non-linear fitting to each time courses. This is because only 5 points seem not to be enough to calculate up to 3 variables accurately, and transcripts which are degraded with a similar kinetics may be regulated in a common mechanism and thus may follow the same dynamics. We removed time course data whose tag counts in 0 hour after ActD addition are less than 20, because time courses starting from low number of tags would be unreliable. These criteria resulted in 45496 time course data available in total. Then we calculated ratio of tag counts remaining, namely tag counts were divided by tag count at 0 hour after ActD addition. Since we would like to classify the dynamics of unstable genes, the time course with > 89% (= $2^{-4/24}$; a level which would correspond with a half-life of > 24 hours) of the original RNA level at 4 hour after ActD addition were labeled as long-lived and removed from the further classification. Finally, there were 23445 time course data remaining.

Distance between two time course data $x = (x_0, x_{0.5}, x_1, x_2, x_4)$ and $y = (y_0, y_{0.5}, y_1, y_2, y_4)$, $d(x, y)$, was calculated as $d(x, y) = cos^{-1}$ cor(x, y), where cor(x, y) is Pearson correlation coefficient between x and y. Using the distance metric, we clustered the time course data with density peak clustering algorithm modified as described later. According to the criteria proposed in the paper, we selected 16 cluster centers and then classified all the time course data into 16 clusters.

2) Calculation of degradation rates.

This degradation kinetics can be modeled by a differential equation $dr/dt = \mu - \delta r$, where μ, δ and r represent transcription rate, degradation rate, and RNA level, respectively. Then we have a general solution $r(t) = r_1 + (r_0 - r_1)\exp(-\delta t)$, where r_0 and r_1 $(=\mu/\delta)$ are initial and end points of RNA level, respectively. ActD inhibits activity of RNA PolII, thus ideally μ equals zero when ActD is added, and this leads to the model 1, $r_0 = 1$ and $r_1 = 0$ with varying δ. However, if the inhibition by ActD is not complete, μ does not equal zero and thus $r_1 > 0$. The model 2 where $r_0 = 1$ with varying r_1 and δ, represents this situation. The model 3, $r_1 = 0$ with varying r_0 and δ, has two meanings. It is possible that the tag counts at 0 hour after ActD addition are fluctuated by observation through RNA-seq. This leads to varying r_0. The other possible reason where r_0 is not equal to 1 is the delay in the inhibition of transcription by ActD. Suppose ActD starts to inhibit transcription at time τ after addition, then we have $dr/dt = \mu \cdot u(\tau - t) - \delta r$, where $u(t)$ is Heaviside step function, $u(t) = 0$ or $u(t) = 1$ if $t < 0$ or $t > 0$, respectively. Given $r_0 = \mu/\delta$, this equation has a solution $r(t) = r_0$ or $r(t) = r_0\exp(-\delta(t-\tau))$ if $t<\tau$ or $t>\tau$, respectively. In this case the fitted r_0 will be effectively equal to $r_0\exp(\delta\tau)$ which can take non-unit value $\exp(\delta\tau)$ depending on δ and τ. The most complex model considered, model 4, is derived

from the combination of these effects.

To estimate the parameters, nonlinear least-square fitting was performed for each clusters by using modified Levenberg-Marquardt method with some modification[46]. Only "core" of the clusters were used for the calculation. Then models were selected according to Akaike's Information criterion (AIC) modified for small number of data points, namely, $AIC = n\log RSE + 2np/(n-p-1)$, where n, RSE, and p are number of data points used for fitting, residual standard error, and number of parameters, respectively[24]. The calculation resulted in 16 clusters having their own r_0, r_1, and δ.

3) Classification of genes by using time course of degradation rates.

Next we classified the genes according to the dynamics of degradation rates. Each gene has 8 time points, and each time points were classified into one of 10 clusters defined above plus "long-lived" or "NA" (which represents time points not used for calculation). The resulting data looks like (C^g_0, $C^g_{0.5}$, C^g_1, C^g_2, C^g_3, C^g_4, C^g_6, C^g_8) for a gene g, where $C^g{}_t$ is the cluster in which the gene g belongs to at t = 0, 0.5, 1, 2, 3, 4, 6 and 8 hours after LPS addition. The distance metric between two genes g and h, $D(g,h)$, was defined as $D(g,h) = \Sigma_t\, d(m(C^g_t), m(C^h_t))$, where $m(C)$ represents mean of the time course of genes in cluster C, and d is defined as above, namely $d(m(C^g_t), m(C^h_t)) = cos^{-1}$ cor($m(C^g_t)$, $m(C^h_t)$). To apply the distance to data containing "NA", the distance between "NA" and any cluster C including "NA" and "long-lived" defined as d("NA", C) = d_{median}, where d_{median} is median of all the distances in between clusters other than "NA" or "long-lived". In case where "long-lived" is included in the data, we define d("long-lived", C) = 0, namely, no correlation, for any C other than "NA" or "long-lived", and d("long-lived", "long-lived") = 1.

Density peak clustering method with the metric was utilized and number of cluster centers was determined as 10. The result is shown in Addtional file 3.

4) Modified density peak clustering.

We utilized a clustering algorithm proposed by Rodirguez and Laio[23], called “density peak clustering” in the current report. Briefly, local density of points, ρ, minimum distance to the point with higher density, δ, and their product $\gamma= \rho\delta$, are calculated, and “cluster centers” are identified as points having high γ. Distance between the cluster centers and remaining points are calculated, and remaining points are assigned to the same cluster as the nearest point of higher density. Then, a border region of a cluster is defined as the set of points which is “close” to the other cluster. A “halo” of cluster is defined as a point which has density lower than the highest density in the border region ρ_b. The remaining points in the cluster are defined as a “core”.

We changed the procedure written in Rodriguez and Liao as follows: We determined the local density with a *k*-nearest neighbor method. In our analysis k=150 and k=50 in the first and the second clustering, respectively, briefly following a rule of thumb where k is set to square root of the number of points in the dataset. Also, we defined a border density of a cluster as the set of points whose *k*-nearest neighbor has points in other clusters. In our analysis we set k = 12 and 10 in the first and the second clustering, respectively. Then we defined ρ_b as a mean of densities of the border region points. By these modifications we could perform the clustering in a non-parametric manner.

Detailed procedure for de novo RNA secondary structure motif prediction.

We first cut the 3’ UTR sequences in a cluster of interest into bins of 100 bases long with 50 bases overlap between adjacent bins. This procedure helps to avoid small but conserved and functional elements to be ignored by long but non-conserved and presumably occasional alignments, which typically has high alignment score and thus tends to be picked up more frequently than the small functional elements. Next, we randomly selected the bins from each gene and generated sets of bins up to 700 times (due to limitation on computational resource). Pairwise alignment by using Foldalign[28] was performed on those at most 700 sets of sequence bins. In this step we limited the length of alignments to less than 40 bases, and difference of length is less than 5. To filter out non-conserved hits, phastCons scores for the aligned sequences were calculated and alignments with sequences having mean score of less than 0.7 were removed. As described in the main text, since alignment scores and lengths of the alignment are correlated, we selected top scored alignments from each length to avoid selecting only long alignments. Having wide range of length is crucial because known structure motifs have length ranged from around 15 (Roquin and Regnase-1)[37, 38] to around 40 (AUF1)[43]. After selecting alignments of top 0.2 % score as “seeds”, we performed building of SCFG model[29] by using cmbuild program in Infernal[30]. All the models were calibrated by using cmcalibrate program. Then we searched for motifs in the cluster by using cmsearch program with the models. Search hits with E value of less than 1 were aligned and subjected to building of models. This cycle was performed until no new sequence hits were found on the search, or if length of the new model was more than 5 bases longer than the original. The latter condition prevents the model to be too long. After building the models, we searched for the motif in all the 3’ UTRs, and numbers of hits for each motif were subjected to over-representation analysis.
