# Supplementary figures and images for: Genome-wide map of RNA degradation kinetics patterns in dendritic cells after LPS stimulation facilitates identification of primary sequence and secondary structure motifs in mRNAs
Source: BMC Genomics. 2016 Dec 22;17(Suppl 13):1032. doi: 10.1186/s12864-016-3325-7 (PMC5259865; doi:10.1186/s12864-016-3325-7)

## Additional File 4.

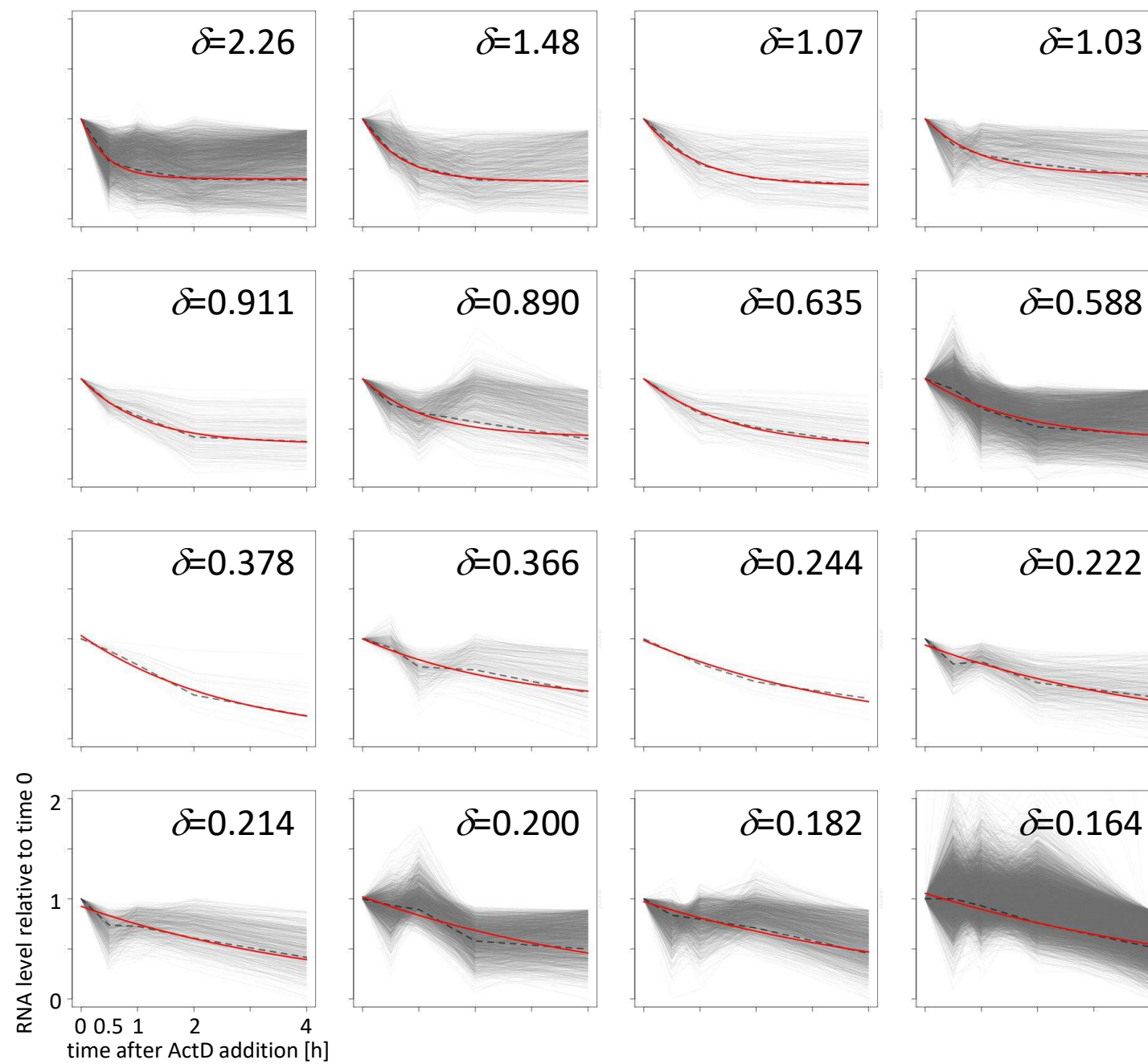

Supplement: Additional file 3: — Pattern recognition of degradation kinetics. Time course data in each of 16 clusters with the mean of the core data of the cluster (black dashed line) and fitting curve (red line) were plotted and shown. The clusters are in the descending order of degradation rates calculated from the fitting, from top, left to right, to bottom. (PDF 443 kb) [file 12864_2016_3325_MOESM3_ESM.pdf]

## Additional File 5.

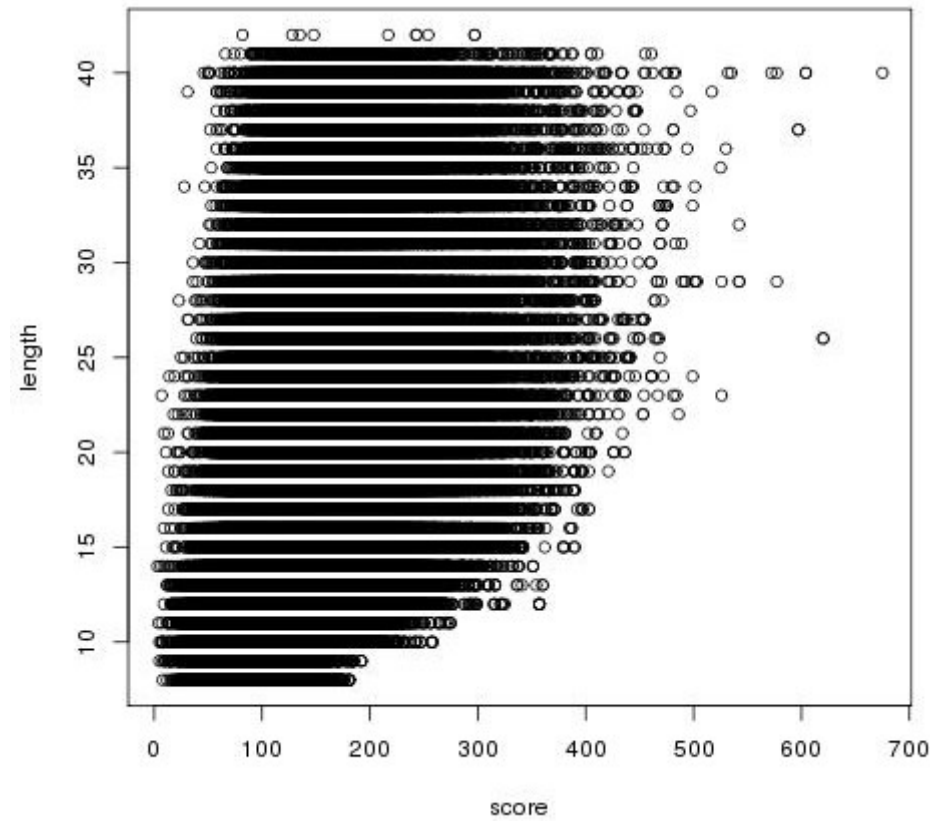

Supplement: Additional file 5: — Scatter plot of alignment scores vs alignment lengths obtained from Cluster I. Scores and lengths of conserved alignments from Cluster I were plotted and shown. (PDF 238 kb) [file 12864_2016_3325_MOESM5_ESM.pdf]
