# Supplementary material for: Genome-wide map of RNA degradation kinetics patterns in dendritic cells after LPS stimulation facilitates identification of primary sequence and secondary structure motifs in mRNAs
Source: BMC Genomics. 2016 Dec 22;17(Suppl 13):1032. doi: 10.1186/s12864-016-3325-7 (PMC5259865; doi:10.1186/s12864-016-3325-7)
Supplement: Additional file 6: — Multiple alignment of sequences found with each motif. Sequences found by searching with the motifs were aligned by Infernal and shown in Stockholm format. Refseq ID of the origin of the sequence, positions of the 3′ UTR in the mRNAs of the Refseq ID, and positions of the sequence found by motif searching was shown in the left column. Common secondary structure (SS_cons) and consensus sequence (RF) are also shown. (PDF 214 kb) [file 12864_2016_3325_MOESM6_ESM.pdf]

Additional file 6.

motif.0.271

NM_013693_876-1653/545-561	UGUUUUCUGUGAAAACG
NM_008965_2032-3264/413-429	UCUUCUCUGUGAGAAGG
NM_001163818_3200-4239/888-904	UGUUUUCUAUGAAAACA
NM_172142_1452-2010/214-230	UGAUUUCUGUGAAAUCG
NM_183148_1388-5352/979-995	AUUUCUCUGUGAGAAAG
NM_145968_2309-3076/99-115	UCUCCUCUGUGAGGAUG
NM_133662_490-1090/563-579	UAUGUUCUGUGAACACG
NM_001172205_1874-5699/1929-1945	UUCCUUCUGUGAAUGAG
NM_011893_1803-2966/1021-1037	CUUUUUCUGUGAAAAUA
NM_026268_1608-2814/378-394	UGUCUUCAGUGAAGACU
NM_001025312_1760-4143/1516-1532	UCUUCUCUAUGAGAAUA
NM_001081049_11893-16439/2405-2421	UUUUUUUUAUGAAAAAA
NM_019835_1314-4205/2858-2874	UUUUUUCAGUAAAAAAU
NM_010234_1283-2107/619-635	AGUUUUCCAUGAAAACG
SS_cons	<<<<<<_____>>>>>>
RF	uuUUUUCUGUGAAAAag

motif.0.170

NM_013693_876-1653/700-715	UGCUCCCCACG.GGAGC
NM_177412_2251-5719/3127-3142	UGGUUCCCACA.GCACC
NM_178707_3994-7292/963-978	UGCUGCCCACG.GGAGC
NM_007570_580-3083/1266-1281	UGGUGCUCAGA.GCACC
NM_007707_1259-2742/1108-1123	UGCCGCUCACA.GGGGC
NM_198103_2231-7427/4747-4762	AGCUUCCCAAA.GCAGC
NM_010638_1265-3263/3-18	UGCUCCCCAUG.GCAGC
NM_016780_2454-5795/3207-3222	AGCUGCCCACA.GCAAC
NM_021493_4176-5450/558-574	UGGUGCACACAgGCACC
NM_133662_490-1090/487-503	UGCUUCUCAGGgGCAGC
NM_017373_1724-2019/174-189	AGCCCCCCACA.GAGGC
SS_cons	:<<<<<____.>>>>>
RF	UGCUgCcCAcA.GcAGC

motif.0.107

NM_001177464_3722-7703/1768-1788	AUAUACAUAUAUAUAUAUAUA
NM_001164598_1714-4530/68-88	AUACAUAUAUAUAUAUAUAUA
NM_001177464_3722-7703/1736-1756	AUAUAUAUAUAUACAUAUAUA
NM_007625_1877-5213/365-385	AUAUAUAUACAUAUAUAUAUA
NM_001163502_4033-7266/3020-3040	GUACAUAUAUAUAUAUAUAUA
NM_020575_2244-2719/161-181	AUAUAUAUAUAUACAUGUAUA
NM_198035_2309-5970/1714-1734	AUAUAUAUAUAUAUAUAUAUA
NM_007548_2713-5221/203-223	AUAUACAUAUAUACAUAUAUA
NM_009744_2213-3326/478-498	AUAUAAAUAUAUAUAUAUAUA
NM_001081049_11893-16439/3668-3688	AUAUAAAUAUAUAUAUAUAUA
NM_153153_6564-7424/351-371	UUCUAAAUAUAUAUAUAUAUA
NM_007746_1529-2507/837-857	AUAUAUAUAUGCAUAUAUAUA
NM_153088_1076-2790/693-713	AUAUAAAUAUGUAUAUGUGUA
NM_001162921_2713-6133/3377-3397	AUGUAUCUAUAUGUAUAUAGA
NM_023502_2260-5900/558-578	UUUUGAAUAUAUAUAUAUAUA
NM_019763_11284-12299/649-669	AUGUGUAUAUAUAAAUAUAUA
NM_172911_4411-4780/108-128	UUUUAUAUAUAUACAUAUAUA
NM_019921_2171-3879/1310-1330	UUUUAAAUAUAUAUAUAUAUG
NM_008839_3263-8917/4825-4845	GUAUGUGUAUAUAUAUAUAUA
NM_177412_2251-5719/673-693	UUUUAUAUAUAUAUGUGUAUA
NM_198035_2309-5970/1678-1698	AUACAUACAUAUAUGUAUGUA
NM_138679_8978-11293/1108-1128	GUAUAAAUAUAUAUAUGUGUG
NM_172569_2506-3763/55-75	GUAUAUAUAUGUAUGUAUGUA
NM_138679_8978-11293/1136-1156	GUGUAUAUAUAUAUAUGUGUG
NM_178707_3994-7292/3249-3269	UUAUAAAUAUGUAUAUUUAUA
NM_001025067_3331-7134/1340-1360	AAGUAACUGUAUAUAUACAGA
NM_138679_8978-11293/1164-1184	AUGUGUAUAUAUAUAUGUGUG
NM_001004176_4272-6514/2209-2229	AUAUAAAAAUAUAUAUAUAUA
NM_172569_2506-3763/79-99	AUGUACGUAUGUAUGUAUAUG
NM_198035_2309-5970/1648-1668	CUAUACAUAUAUAUGUGUGUG
SS_cons	::::::<<<<<____>>>>>:
RF	AUAUAUAUAUAUAUAUAUAUA
